# Supplementary material for: Co-Transplantation of Barcoded Lymphoid-Primed Multipotent (LMPP) and Common Lymphocyte (CLP) Progenitors Reveals a Major Contribution of LMPP to the Lymphoid Lineage
Source: Int J Mol Sci. 2023 Feb 22;24(5):4368. doi: 10.3390/ijms24054368 (PMC10002536; doi:10.3390/ijms24054368)
Supplement: Supplementary file 1 [file ijms-24-04368-s001.zip › lopez_et_al_suppFiguresCaptions.pdf]

Table S1: Sequences of barcodes and their specific primers. The sequence for all barcodes are shown. The identical 5' and 3' ends are in bold. The primers used for the specific amplification of each barcode are shown in the right column.

Table S2: CLP and LMPP distribution into mirror mice.

For each sample, described in **Figure 8**, are detailed cell population, its ID, mouse ID, BC group (LMPPA + CLP B or inversely), total read number, percentage of reads aligned exactly 1 time on barcode genomic sequence, proportion of counts per barcode (B1, B4, B6, B12, B25, B2, B3, B7, B10, B15), and proportion of counts per cell type (CLP or LMPP). Raw counts, CPK counts and mix normalised value for each barcode in each sample are detailed. For each calculated value (proportion of counts, CPK counts, mix normalised value), the corresponding formula is also detailed.

Figure S1: Gating strategies for barcoded LMPP and barcoded CLP sorting. BM cells were labeled for lineage (NK1.1, TCRβ, CD3e, CD11b, CD19, Ly-6G, and Ly76) markers, c-Kit, Sca-1, VCAM-1, Flt3, and IL-7Ra. *In vivo* transduced LMPP and CLP progenitors were obtained after gating on the GFP expression. LMPP represented 31.2% ± 3.4 and CLP 30.1% ± 11.1 in their respective windows (mean of 8 experiments). Numbers indicate the percentage of population in the respective window.

Figure S2: Reconstitution potential of HSC and LMPP/CLP in absolute numbers. Evaluation of the myeloid lineage CD11b+ in the BM (**A**), B cell lineage in the BM and spleen (**B**) and T cell lineage in the spleen and thymus (**C**) of mice grafted with HSC (empty dots), or LMPP/CLP progenitors (triangle). Black dots represent the indicated lineages at the steady state in normal unmanipulated mice. Each dot represents one mouse.
