# Supplementary figures and images for: Co-Transplantation of Barcoded Lymphoid-Primed Multipotent (LMPP) and Common Lymphocyte (CLP) Progenitors Reveals a Major Contribution of LMPP to the Lymphoid Lineage
Source: Int J Mol Sci. 2023 Feb 22;24(5):4368. doi: 10.3390/ijms24054368 (PMC10002536; doi:10.3390/ijms24054368)

Figure S1

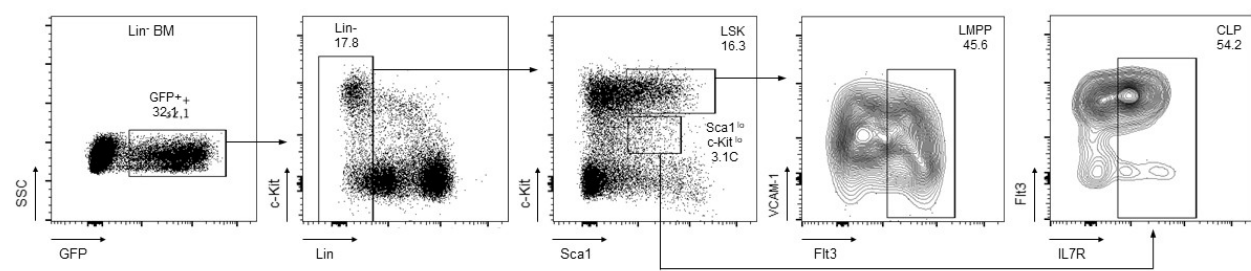

Figure S2

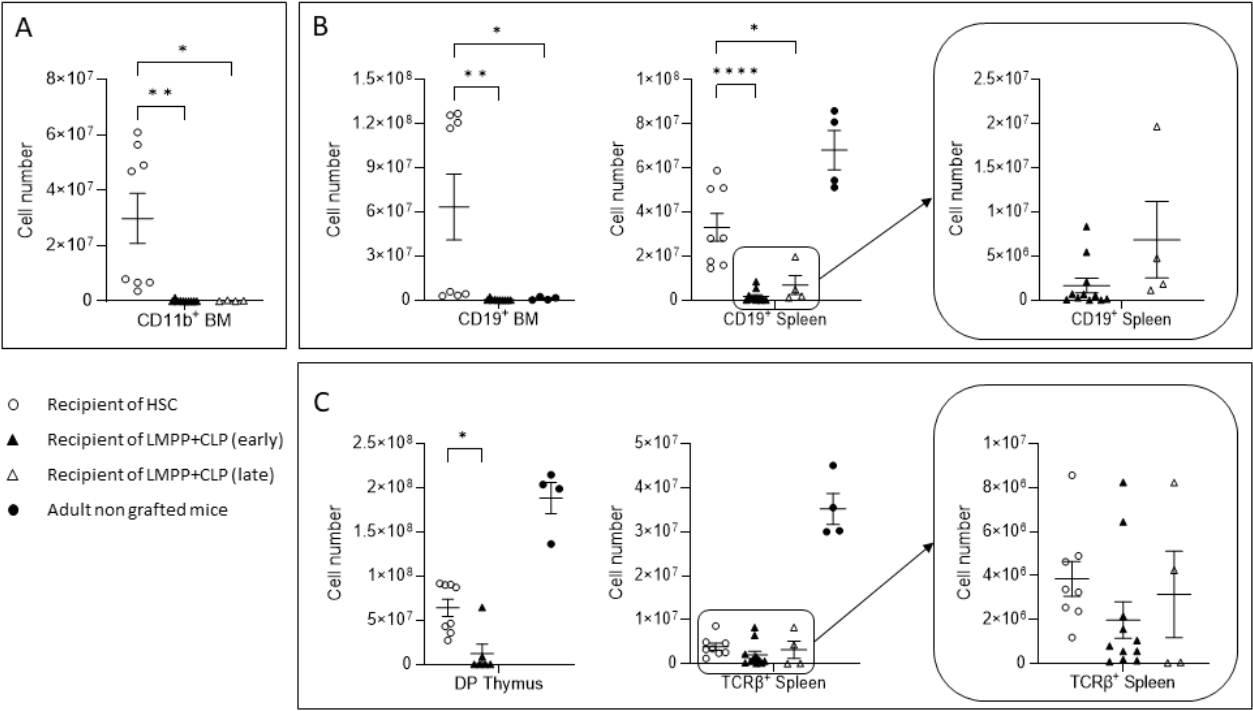

Supplement: Supplementary file 1 [file ijms-24-04368-s001.zip › lopez_et_al_suppFigures.pdf]
